# Diagnostic Accuracy of the Three‐Gene Host‐Response Test in Blood for Tuberculosis: A Systematic Review

**DOI:** 10.1155/ijm/3402245

**Published:** 2026-11-06

**Authors:** Carlos Quispe-Vicuña, Dante Julca-Marín, Milagros Pascual-Guevara, Fabian A. Chavez Ecos, Carlos J. Zumaran-Nuñez, Fritz Fidel Váscones-Román, Carlomagno Villarreal-Inca, Miguel Cabanillas-Lazo, Luz Quispe-Garate, Elizabeth F. Hidalgo Ferrer, J. Smith Torres-Roman, Julio A. Poterico, Miguel A. Arce-Huamani, Vicente Benitez-Zapata

**Affiliations:** ^1^ NEMECS Research Group: Neuroscience, Metabolism, Clinical and Health Effectiveness Universidad Científica del Sur Lima Peru cientifica.edu.pe; ^2^ Sociedad Científica de San Fernando Lima Peru; ^3^ Facultad de Medicina Universidad Nacional Mayor de San Marcos Lima Peru unmsm.edu.pe; ^4^ Universidad de Huánuco Huánuco Peru; ^5^ Universidad Señor de Sipán Chiclayo Peru; ^6^ Departamento de Neurocirugía Instituto Nacional de Ciencias Neurológicas Lima Peru; ^7^ Harvard T.H. Chan School of Public Health Harvard University Boston Massachusetts USA harvard.edu; ^8^ Facultad de Medicina Universidad Peruana Cayetano Heredia Lima Peru upch.edu.pe; ^9^ Graduate School Universidad Continental Lima Peru ucontinental.edu.pe; ^10^ Public Health Surveillance and Applied Research Programa Academico de Medicina Humana Facultad de Ciencias de la Salud Universidad Privada Norbert Wiener Lima Peru anahuac.mx; ^11^ Universidad Científica del Sur Lima Peru cientifica.edu.pe

**Keywords:** diagnosis, genetic screening, systematic review, triage, tuberculosis

## Abstract

**Purpose:**

Molecular diagnostic tests and sputum‐based assays are common techniques for the initial evaluation of tuberculosis (TB). However, given their limitations in certain clinical contexts, efforts have been made to develop other diagnostic markers. This review evaluated the diagnostic accuracy of a three‐gene host‐response test (TB score) for the diagnosis of TB.

**Methods:**

We performed systematic searches in seven databases until July 2026. We included observational studies evaluating the diagnostic accuracy of the TB score to diagnosis of TB across different clinical contexts. Risk of bias was assessed using QUADAS‐2. Meta‐analysis was performed using the logit function, and GRADE was employed to assess certainty of evidence (CoE).

**Results:**

Twenty‐eight studies were included. For distinguishing pulmonary TB from other respiratory diseases, the TB score showed a pooled sensitivity and specificity of 0.80 (95% CI: 0.73–0.84) and 0.72 (95% CI: 0.64–0.78), respectively, with an AUC of 0.82. When comparing pulmonary TB to healthy controls, sensitivity was 0.75 and specificity was 0.86, with an AUC of 0.87. Performance was lower for meningeal tuberculosis (sensitivity 0.58; specificity 0.67) and for distinguishing active from latent TB (sensitivity 0.73; specificity 0.76). Risk of bias was generally low. The CoE for most outcomes was very low.

**Conclusion:**

The TB score shows moderate diagnostic accuracy for pulmonary TB, with pooled sensitivity below the WHO TPP minimum for a standalone triage test. High heterogeneity and very low CoE limit confidence in these findings. Its potential role as a complementary tool remains investigational and requires validation in prospective studies.

## 1. Introduction

Tuberculosis (TB) is an infectious disease caused by *Mycobacterium tuberculosis* that remains a major global health problem, with 8.4 million cases and 1.2 million deaths reported in 2021 [1, 2]. Despite diagnostic advances, early detection remains challenging, particularly in patients with low bacillary burden, extrapulmonary disease, or immunosuppression, where conventional methods show reduced sensitivity [3–5]. Current diagnostic tools, including sputum microscopy, culture, and tuberculin skin testing, present limitations such as delayed results and suboptimal performance in these populations [3, 4, 6].

In this context, host‐response biomarkers have emerged as promising alternatives. In 2016, Cepheid (Sunnyvale, California, United States) developed a prototype three‐gene host‐response blood test that quantifies mRNA expression of guanylate‐binding Protein 5 (*GBP5*), dual specificity Phosphatase 3 (*DUSP3*), and krüppel‐like Factor 2 (*KLF2*) using the GeneXpert platform, generating a composite TB score to distinguish active TB from other conditions [7–9]. More recently, studies have proposed replacing KLF2 with TATA‐box binding protein (*TBP*), showing improved stability and potentially better diagnostic performance [10–12]. This approach offers a rapid, nonsputum‐based diagnostic option, particularly useful in patients unable to produce sputum or with smear‐negative disease. In this regard, previous systematic reviews have evaluated the utility of this TB score [13, 14]; important gaps remain. Prior reviews did not include recent studies incorporating *TBP*‐based signatures, did not assess the certainty of the evidence (CoE), and lacked analyses of subgroups (for example, comparing TB with other respiratory diseases [ORD] or healthy controls [HC]). Therefore, an updated synthesis of the evidence is needed to better define the clinical utility of this biomarker.

The results of this systematic review would allow for a comprehensive evaluation of the diagnostic utility of the TB score, providing valuable information for clinical decision‐making regarding the diagnosis of tuberculosis, which is particularly relevant in the current global context given the growing burden of tuberculosis. For all this, our objective was to determine the diagnostic accuracy of the TB score.

## 2. Methods

### 2.1. Review Protocol and Registration

This systematic review was conducted following the methodology outlined in the Cochrane Handbook [15] and the “Preferred Reporting Items for Systematic Reviews and Meta‐Analyses of Diagnostic Test Accuracy Studies” (PRISMA‐DTA statement) (Table S1) [16]. The study protocol was registered in PROSPERO (Code: CRD420251157589).

The clinical question was “What is the accuracy of three‐gene host‐response test in blood for detecting tuberculosis?” This question was structured according to the following format: (1) population, patients with symptoms suggestive of TB; (2) index test, three‐gene host‐response blood test; (3) reference test, sputum test using Xpert Ultra and/or cultures; and (4) outcome, sensitivity, specificity, area under the curve (AUC), predictive values, and diagnostic odds ratio (DOR).

Studies using culture, Xpert Ultra, or both as reference standards were eligible for inclusion and were combined in the meta‐analysis. Differences in the reference standard were subsequently explored through subgroup analysis.

### 2.2. Search Strategy

We performed systematic search in PubMed, Embase, Scopus, LILACS, Cochrane Central, and Web of Science until July 5, 2026. The search strategy was developed for PubMed and adapted for use in other databases, incorporating key terms related to “*Cepheid 3-gene*” and “*tuberculosis*”. The search strategy is reported in Table S2. The searches were completed by reviewing the bibliographic references of the included studies and selecting the articles that met the requirements. We also conducted a search for gray literature using Google Scholar.

### 2.3. Screening and Eligibility Evaluation

The electronic search results were imported into Endnote X9, where duplicate records were automatically removed. These records were exported to the Rayyan (https://rayyan.qcri.org/) [17].

We included observational diagnostic studies (cross‐sectional, case–control, and cohort) that evaluated the PICO components. No restrictions were applied regarding language or publication date. Reviews, letters, editorials, and no full‐text articles were excluded.

For study selection, a peer review process was conducted by four reviewers (C.Q‐V., D.J‐M., C.V‐I., and L.Q‐G.), who independently applied the inclusion criteria in two steps. First, titles and abstracts were screened, followed by a full‐text review of studies selected in the initial step. Any discrepancies were resolved by consensus with another author (V.B‐Z.).

Interreviewer agreement was high for both title/abstract screening (*κ* = 0.94) and full‐text assessment (*κ* = 0.95) (Table S3).

### 2.4. Data Extraction

Four authors (M.P‐G., F.A.C‐E., C.J.Z‐Ñ., F.V‐R.) independently extracted data from each study in a blinded manner. Any disagreements were resolved by a consensus with a fifth author (V.B‐Z.). For each study, data was extracted on the author, year, country, design, total population, HIV (%) in TB population, diabetes Type 2 (DM2) (%) in TB population, sample used (capillary blood, venous blood, or whole blood), mean age, male gender (%), type of tuberculosis (sensitive, MDR, or XDR), comparation, genetic signature, cutoff value, and reference test.

We also extracted data on diagnostic accuracy from the studies, including sensitivity, specificity, predictive values, and DOR. In addition, data were collected on the number of true positives (TP), true negatives (TN), false positives (FP), and false negatives (FN). If a study did not provide accuracy results, we calculated these estimates as follows: (1) sensitivity: TP/(TP + FN); (2) specificity: TN/(TN + FP); (3) positive predictive value: TP/(TP + FP); (4) negative predictive value: TN/(TN + FN); (5) LR+: LR + = sensitivity/(1–specificity); (6) LR − = (1–sensitivity)/specificity; (7) DOR: (TP/FN)/(FP/TN); (8) summary ROC (SROC) curve; (9) AUC; and (10) Fagan nomogram. If a study did not provide sensitivity and specificity values, they were calculated manually.

The extracted data were grouped into four comparisons to better assess the diagnostic accuracy of the TB score: (1) pulmonary TB vs. ORD; (2) pulmonary TB vs. HC; (3) extrapulmonary meningeal tuberculosis vs. other neurological diseases; and (4) active TB vs. latent TB.

### 2.5. Risk of Bias (RoB) and Applicability

The RoB of the studies was assessed with the revised tool for the quality assessment of diagnostic accuracy studies (QUADAS‐2) [18] by two authors (M.P‐G. and C.Q‐V.) independently. Any disagreements were resolved by a consensus with a third author (V.B‐Z.). This tool evaluates four domains: (1) patient selection; (2) index test; (3) reference standard; and (4) flow and timing.

### 2.6. Subgroup Analyses

To explore potential sources of heterogeneity, we conducted subgroup analyses comparing pulmonary TB vs. ORD by (1) age group (children vs. adults); (2) reference standard (culture, GeneXpert Ultra, or both); (3) HIV serostatus (positive vs. negative); (4) economic‐geographic status (high‐income regions vs. low‐ and middle‐income regions according to World Bank reports [19]); (5) genetic signature (*GBP5*, *DUSP3*, and *KLF2* vs. *TBP*), (6) tuberculosis burden (high vs. low, according to WHO TB reports [20]); (7) RoB (high vs. low), and (8) study design (cross‐sectional/cohorts vs. case–control). In addition, a statistically significant difference (*p* < 0.05) was assessed in each subgroup. Although the two genetic signatures differ in their third gene, they were considered related host‐response tests targeting the same diagnostic purpose and were therefore combined in the primary analysis. Their potential differences were assessed through the subgroup analysis.

### 2.7. Publication Bias

We used a Deeks plot and the Deeks regression asymmetry test to evaluate symmetry but only if the number of studies was greater than 10 studies. We considered *p* < 0.05 as presence of significant asymmetry and publication bias.

### 2.8. Summary of Finding (SoF) and CoE

Results were summarized by outcome and intervention in a SoF table.

Two authors (C.Q‐V. and M.C‐L.) assessed the CoE synthesis by applying the GRADE (Grading of Recommendation, Assessment, Development, and Evaluation) system to diagnostic accuracy [21, 22]. This assessment is based on five domains: RoB, imprecision, indirectness, inconsistency, and publication bias, as outlined in the GRADE manual [23]. The GRADE process assigned an initial quality level based on study design (cross‐sectional and cohort studies were considered high quality), subsequently applying specific downgrading factors to determine the level of evidence. The CoE was reported as high, moderate, low, or very low.

In addition, for the calculation of the absolute effect per 1000 patients analyzed, we estimated a prevalence of 33%, which was calculated by synthesizing the TB prevalence data from the included studies.

### 2.9. Statistical Analysis

In the qualitative synthesis, we described studies based on key characteristics and findings. For quantitative synthesis, we conducted meta‐analyses whenever data allowed, focusing on pooled estimates of sensitivity and specificity. Sensitivity and specificity were pooled separately using random‐effects models with a logit transformation (PLOGIT), and 95% confidence intervals were calculated using the Clopper–Pearson method. In addition, a bivariate random‐effects model (Reitsma) was used to jointly model sensitivity and specificity and to construct the SROC curve and estimate the AUC. The separate random‐effects models were used to obtain pooled clinical diagnostic accuracy estimates, whereas the bivariate model was used to account for the correlation between sensitivity and specificity and to assess diagnostic discrimination through the SROC curve and AUC. A leave‐one‐out sensitivity analysis was used to understand the impact of each individual study on the pooled effect estimate.

We assessed statistical heterogeneity between studies using the *I* ^2^ statistic, categorizing it as low (*I* ^2^ < 30*%*), moderate (30%–60%), and high (*I* ^2^ > 60*%*). To complement this, we visually inspected forest plots to corroborate the degree and pattern of heterogeneity, observing overlapping confidence intervals and any potential outliers. If heterogeneity is significant (> 75%), a metaregression analysis was conducted to identify the source of heterogeneity. In addition, 95% prediction intervals (PI) were calculated for each outcome using Higgins′s method [24] using the predict() function. The analysis was conducted using R Studio Version 4.3.3 [25].

## 3. Results

### 3.1. Study Selection

We identified 722 studies through our systematic search. After selection, 28 studies [7–12, 26–47] were finally included (Figure 1). A complete list of excluded articles is provided in Table S4.

**Figure 1:**
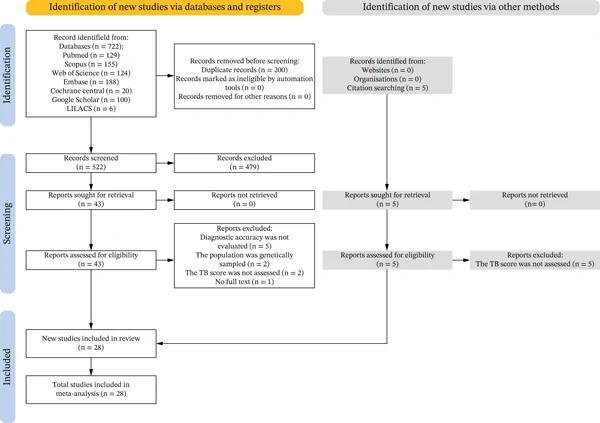
PRISMA 2020 flowchart of included studies.

### 3.2. Characteristics of Included Studies

Of the 28 included studies, most used a cohort design (16 studies). Most of the studies were conducted in China (eight studies). The mean age was 36.2 ± 25.4 years, with a slight male predominance (55.1%). This population was heterogeneous and included pediatric populations and HIV‐positive patients, among others. Regarding the index test, 22 studies evaluated the three‐gene signature (*GBP5*, *DUSP3*, and *KLF2*), primarily employing whole blood or venous blood samples. Reference standards primarily combined liquid sputum culture and Xpert Ultra into a composite microbiological standard. Most studies focused on pulmonary TB, whereas Huynh et al. 2024 [33] and Huynh et al. 2026 [34] evaluated extrapulmonary TB (tuberculous meningitis). Most of the studies did not specify whether the study populations consisted of cases of drug‐sensitive or drug‐resistant TB. Overall, AUC values ranging from 0.53 to 0.94 were reported (Table 1 **)**. Of the 28 included studies, 27 [7, 9–12, 26–47] were included in the meta‐analysis (17,692 participants) because Sutherland 2022 [8] reported preliminary results later published by Sutherland 2025 [41].

**Table 1:** Characteristics of included studies (*n* = 28).

Study	Country	Design	Total population	HIV (%) in TB population	DM2 (%) in TB population	Sample used	Sex male, (%)	Age, mean ± SD (years)	Comparation	Genetic signature	Reference test	Cutoff value	AUC
Chendi et al., 2023	Norway	Cohort	31	0	NR	Venous blood	48.4	50.6 ± 16.4	TB vs. non‐TB‐LRI	GBP5, DUSP3, and KLF2	Culture	NR	0.86
Crowder et al., 2026	Philippines, Vietnam, Uganda, South Africa, and India	Cohort	1392	14	14	Capillary or venous blood	54.5	43 ± 16	TB vs. non‐TB	GBP5, DUSP3, and TBP	Xpert Ultra or culture	≤ −1.5	≤ −1.5: 0.91 and ≤ −1.3: 0.89
Folkesson et al., 2025	Sweden	Cohort	312	NR	5.6	Capillary or venous blood	52.2	50.67 ± 52.59	TB vs. non‐TB	GBP5, DUSP3, and TBP	Culture	−1.25	0.84
Francisco et al., 2017	China	Cohort	562	0	NR	Whole Blood	55.7	46.13 ± 45.19	Active TB vs. (ORD, HC)	GBP5, DUSP3, and KLF2	Culture	NR	TB vs. ORD: 0.71 and TB vs. HC: 0.85
			855			PBMC	60.8	47.57 ± 46.67	Active TB vs. (ORD, HC)	GBP5, DUSP3, and KLF2	Culture	NR	NR
Gupta‐Wright et al., 2024	Vietnam, India, the Philippines, Uganda, and South Africa	Cross‐sectional	1499	15.3	13.1	Venous blood	54.2	41.17 ± 18.52	TB vs. non‐TB	GBP5, DUSP3, and TBP	Xpert Ultra	NR	0.89
Huynh et al., 2024	Vietnam	Cross‐sectional	791	39	NR	Venous blood	69	40.83 ± 15.98	TB meningitis vs. other neurologic onfections	GBP5, DUSP3, and KLF2	Xpert Ultra	NR	0.66
Ji et al., 2024	China	Cross‐sectional	297	1.68	4.04	Venous blood	55.2	50.33 ± 19.26	TB vs. non‐TB	GBP5, DUSP3, and TBP	Xpert Ultra or culture	NR	0.82
Li et al., 2023	China	Cross‐sectional	590	0	NR	Venous blood	63.7	57.67 ± 13.33	TB vs. (ORD, HC)	GBP5, DUSP3, and KLF2	Xpert Ultra or culture	NR	TB vs. HC: 0.912 and TB vs. ORD: 0.798
Moreira et al., 2021	Brazil	Case–control	300	4	NR	Whole blood	100	32.67 ± 8.15	Active TB vs. non‐TB	GBP5, DUSP3, and KLF2	Xpert Ultra or culture	NR	0.84
Olbrich et al., 2024	Malawi, Mozambique, South Africa, and Tanzania	Cohort	639	8	NR	Whole blood	52	5.47 ± 5.48	Confirmed TB vs. nonconfirmed TB	GBP5, DUSP3, and KLF2	Xpert Ultra or culture	1.5	0.85
Seifert et al., 2025	Pakistan	Cohort	497	1	NR	Whole blood	50.9	36.67 ± 19.26	TB vs. non‐TB	GBP5, DUSP3, and TBP	Xpert Ultra or culture	−2.35	0.82
Södersten 2021	South Africa	Case–control	201	100	NR	Whole blood	35	36.33 ± 8.89	TB vs. non‐TB	GBP5, DUSP3, and KLF2	Xpert Ultra or culture	NR	Culture: 0.89 and Xpert: 0.94
Sutherland et al., 2025	South Africa	Cross‐sectional	1262	5.6	6.9	Capillary blood	58.6	34.83 ± 14.81	TB vs. non‐TB	GBP5, DUSP3, and KLF2	Xpert Ultra	≤ −1.275	0.92
Sutherland et al., 2022^a^	South Africa	Cross‐sectional	195	5.3	NR	Capillary blood	62.1	33.67 ± 10.37	TB vs. non‐TB	GBP5, DUSP3, and KLF2	Xpert Ultra or culture	≤ −1.275	0.94
Warsinske et al., 2018	South Africa	Cohort	363	0	NR	Venous blood	51.2	39.00 ± 44.44	TB vs. non‐TB	GBP5, DUSP3, and KLF2	Xpert Ultra or culture	≤ −0.047	0.86
Wu et al., 2023	China	Cohort	653	0	1.3	Whole blood	65.1	50.67 ± 22.96	Active TB vs. (passive TB, ORD, and HC)	GBP5, DUSP3, and KLF2	Culture	NR	Active TB vs. non‐ATB: 0.814, active TB vs. passive TB: 0.739, active TB vs. ORD: 0.825, and active TB vs. HC: 0.892
Zhang et al., 2025	China	Cohort	557	NR	NR	Venous blood	50.8	41.21 ± 18.57	(Active and presumptive TB) vs. non‐TB	GBP5, DUSP3, and KLF2	Culture	NR	0.90
Zhao et al., 2024	China	Cohort	479	NR	NR	Venous blood	57.8	≥ 14 years	Active TB vs. (ORD, HC)	GBP5, DUSP3, and KLF2	Xpert Ultra or culture	NR	Active TB vs. HC: 0.879 and active TB vs. ORD: 0.689
Mann et al., 2024	South Africa	Cohort	707	100	NR	Whole blood	42	32.7 ± 8.9	TB vs. non‐TB	GBP5, DUSP3, and KLF2	Culture	NR	0.79 (0.73–0.85)
Petruccioli et al., 2025	Italy	Case–control	104	NR	NR	PBMC	46.2	43.3 ± 17.0	TB vs. (ORD, HC)	GBP5, DUSP3, and KLF2	Culture	NR	TB vs. HC: 0.70, TB vs. ORD: 0.71, and TB vs. ORD + HC: 0.71
Turner et al., 2020	South Africa	Cohort	181	31	NR	Whole blood	52	36.67 ± 15.56	TB vs. non‐TB	GBP5, DUSP3, and KLF2	Xpert Ultra or culture	NR	NR
Huynh et al., 2026	Vietnam	Cross‐sectional	83	0	NR	Whole blood	65.1	4.10 ± 4.65	TB meningitis vs. other neurologic infections	GBP5, DUSP3, and KLF2	Culture	NR	0.53
Sun et al., 2026	China	Cohort	780	NR	NR	Whole blood	52.4	9.33 ± 5.93	Active TB vs. (ORD, passive TB, and HC)	GBP5, DUSP3, and KLF2	Xpert Ultra or culture	2.675	TB vs. HC: 0.786, TB vs. passive TB: 0.652, and TB vs. ORD: 0.771
Poore et al., 2025	Gambia and Uganda	Cohort	181	30	NR	Capillary or venous blood	47	9.33 ± 5.93	Confirmed TB vs. (unlikely or unconfirmed TB)	GBP5, DUSP3, and TBP	Xpert Ultra or culture	−0.65	Culture: 0.70, Both: 0.61
Hoang et al., 2021	England	Cohort	628	4	6	Whole blood	59.8	40.54 ± 16.21	Active TB vs. ORD	GBP5, DUSP3, and KLF2	Culture	−2.82	0.83
Zhao et al., 2026	China	Cross‐sectional	1128	NR	18.5	Whole blood	63.7	≥ 14 years	TB vs. (ORD, HC)	GBP5, DUSP3, and KLF2	Xpert Ultra or culture	TB vs. ORD: −0.94; TB vs. HC: −0.59	TB vs. ORD: 0.625 and TB vs. HC: 0.846
Muwanga et al., 2024	Ethiopia, Malawi, Namibia, Uganda, South Africa, and The Gambia	Case–control	541	26.1	NR	Whole blood	60.6	33.1 ± 11.5	TB vs. ORD	GBP5, DUSP3, and KLF2	Xpert Ultra or culture	NR	0.79
Larsson et al., 2026	Mozambique, Tanzania, and Zimbabwe	Cohort	2079	36.8	NR	Whole blood	37.8	26.6 ± 18.4	TB vs. ORD	GBP5, DUSP3, and TBP	Xpert Ultra or culture	−1.475	NR

Abbreviations: HC, healthy controls; LRI, low respiratory infections; NR, not reported; ORD, other respiratory diseases; PBMC, peripheral blood mononuclear cell; TB, tuberculosis.

^a^This study provides preliminary data from the 2025 Sutherland study.

### 3.3. Synthesis of Data Analysis

#### 3.3.1. Diagnostic Accuracy of the TB Score in Distinguishing Pulmonary TB From ORD

A total of 23 studies (14,116 participants) evaluated the diagnostic accuracy of the TB score for distinguishing pulmonary TB from ORD, finding a sensitivity of 0.80 (95% CI: 0.73–0.84; *I* ^2^ = 95*%*; 95% PI: 0.42–0.95; very low CoE), a specificity of 0.72 (95% CI: 0.64–0.78; *I* ^2^ = 98*%*; 95% PI: 0.31–0.93; very low CoE) (Figure 2), an AUC of 0.82 (0.77–0.88) (Figure S1), a PPV of 0.60 (95% CI: 0.48–0.71; *I* ^2^ = 98*%*), a NPV of 0.87 (95% CI: 0.79–0.92; *I* ^2^ = 98*%*) (Figure S2), an LR+ of 2.30 (95% CI: 2.22–2.38), and an LR− of 0.38 (95% CI: 0.36–0.40) (Figure S3). The DOR was 9.88 (95% CI: 6.86–14.23; *I* ^2^ = 92*%*) (Figure S4).

**Figure 2:**
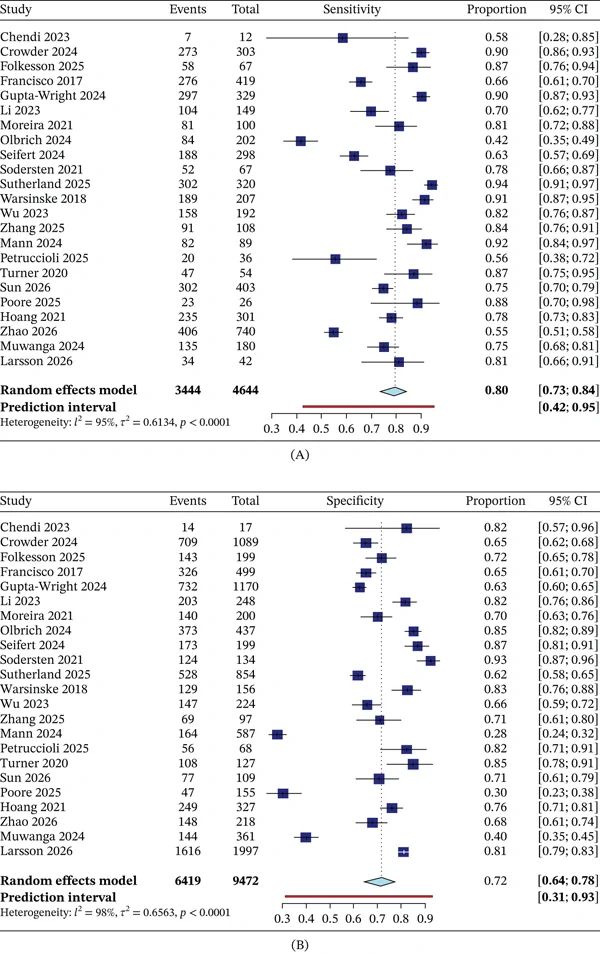
(A) Sensitivity and (B) specificity of TB score in distinguishing pulmonary TB from other respiratory diseases (ORD).

In the subgroup analysis, sensitivity was higher in adults (0.81; 95% CI: 0.75–0.85) than in children with similar specificity. The use of GeneXpert Ultra as a reference showed the highest specificity (0.94; 95% CI: 0.56–0.99). Sensitivity was higher in seropositive populations (0.61; 95% CI: 0.30–0.86) compared with seronegative populations, whereas specificity was comparable. Across all regions, sensitivity was slightly higher in low‐ and middle‐income countries (0.81; 95% CI: 0.74–0.86) than in high‐income settings with similar specificity. When case–control studies were excluded, sensitivity and specificity were comparable with those observed in cross‐sectional/cohort studies. None of the subgroups reported significant differences among themselves. Diagnostic performance was comparable across different genetic signatures and RoB groups (Table 2).

**Table 2:** Diagnostic accuracy of the TB score in distinguishing pulmonary TB from ORD by subgroups.

Subgroups by	*n* studies	Sensitivity		*p*	Specificity		*p*
		Estimate (95% CI)	*I* ^2^		Estimate (95% CI)	*I* ^2^	
Overall	23	0.80 (0.73–0.84)	95%		0.72 (0.64–0.78)	98%	
Age clusters				0.315			0.580
Children	3	0.70 (0.44–0.87)	97%		0.65 (0.35–0.86)	99%	
Adults	20	0.81 (0.75–0.85)	95%		0.73 (0.65–0.79)	98%	
Reference standard^a^				0.823			0.577
Both	16	0.81 (0.71–0.89)	94%		0.79 (0.64–0.89)	97%	
Cultive	9	0.78 (0.66–0.86)	91%		0.83 (0.61–0.94)	98%	
Gene Xpert Ultra	5	0.82 (0.62–0.92)	97%		0.94 (0.56–0.99)	95%	
HIV population^b^				0.558			0.440
Positive	5	0.61 (0.30–0.86)	89%		0.79 (0.45–0.94)	97%	
Negative	20	0.51 (0.41–0.61)	97%		0.66 (0.52–0.78)	98%	
WB regions				0.287			0.224
High income	4	0.73 (0.59–0.84)	79%		0.76 (0.72–0.79)	17%	
Low/middle income	19	0.81 (0.74–0.86)	96%		0.70 (0.61–0.78)	98%	
Genetic signature				0.171			0.584
GBP5, DUSP3, and KLF2	17	0.77 (0.70–0.84)	95%		0.73 (0.64–0.80)	97%	
GBP5, DUSP3, and TBP	6	0.85 (0.76–0.91)	95%		0.68 (0.52–0.81)	98%	
TB burden				0.287			0.224
High	19	0.81 (0.74–0.86)	96%		0.70 (0.61–0.78)	98%	
Low	4	0.73 (0.59–0.84)	79%		0.76 (0.72–0.79)	17%	
RoB				0.199			0.706
High	4	0.74 (0.67–0.81)	66%		0.75 (0.51–0.90)	97%	
Low	19	0.81 (0.74–0.86)	96%		0.71 (0.63–0.78)	98%	
Design				0.199			0.706
Cross‐sectional/cohorts	19	0.81 (0.74–0.86)	96%		0.71 (0.63–0.78)	98%	
Case–control	4	0.74 (0.67–0.81)	66%		0.75 (0.51–0.90)	97%	

Abbreviations: RoB, risk of bias; TB, tuberculosis; WB, World Bank.

^a^Some studies reported diagnostic accuracy results for more than one standard test.

^b^Olbrich et al., 2024 and Larsson et al., 2026 reported the diagnostic accuracy for populations with and without HIV.

In the hypothetical scenario of a 1000‐person cohort, 264 (241–277) patients may have been correctly identified as having pulmonary TB, whereas 66 (53–89) patients with pulmonary TB may have been misclassified as not having pulmonary TB. Additionally, 482 (429–523) people without pulmonary TB may have been correctly classified, whereas 188 (147–241) may have been misclassified as having pulmonary TB (Table 3).

**Table 3:** GRADE summary of findings (SoF).

Diagnostic technique	No. of participants (studies)	Outcome	Effect size (95% CI)	Absolute effect per 1000 patients tested (95% CI)	Certainty of the evidence
Pulmonary TB vs. ORD	14,311 (24 observational studies)	Sensitivity	0.80 (0.73–0.84)	TP: 264 (241–277)	?◯◯◯
				FN: 66 (53–89)	Very low^a,b^
		Specificity	0.72 (0.64–0.78)	TN: 482 (429–523)	?◯◯◯
				FP: 188 (147–241)	Very low^a,b^
Pulmonary TB vs. HC	3724 (seven observational studies)	Sensitivity	0.75 (0.72–0.78)	TP: 248 (238–257)	??◯◯
				FN: 82 (73–92)	Low^b^
		Specificity	0.86 (0.79–0.90)	TN: 576 (529–603)	?◯◯◯
				FP: 94 (67–141)	Very low^a,b^
Extrapulmonary meningeal tuberculosis vs. other neurological diseases	402 (two observational studies)	Sensitivity	0.58 (0.52–0.65)	TP: 191 (172–215)	??◯◯
				FN: 139 (115–158)	Low^a,c^
		Specificity	0.67 (0.46–0.83)	TN: 449 (308–556)	?◯◯◯
				FP: 221 (114–362)	Very low^b,c^
Active TB vs. latent TB	1417 (four observational studies)	Sensitivity	0.73 (0.62–0.81)	TP: 241 (205–267)	?◯◯◯
				FN: 89 (63–125)	Very low^a,b,c^
		Specificity	0.76 (0.68–0.83)	TN: 509 (456–556)	?◯◯◯
				FP: 161 (114–214)	Very low^a,b^

*Note:* GRADE working group grades of evidence. • High certainty: We are very confident that the true effect lies close to that of the estimate of the effect. • Moderate certainty: We are moderately confident in the effect estimate; the true effect is likely to be close to the estimate of the effect, but there is a possibility that it is substantially different. • Low certainty: Our confidence in the effect estimate is limited: The true effect may be substantially different from the estimate of the effect • Very low certainty: We have very little confidence in the effect estimate: the true effect is likely to be substantially different from the estimate of effect.
?: One point out of four given. ◯: No point given.

Abbrevations: CI, confidence interval; FN, false negative; FP, false positive; TN, true negative; TP, true positive.

^a^Imprecision: 95% CI ranges from 10% to 20%. Therefore, we decided to reduce the level of certainty by one level.

^b^Inconsistency: *I* ^2^ > 60*%* was detected in meta‐analyses. Therefore, we decided to reduce the level of certainty by two levels.

^c^Imprecision: Effect size does not exceed the imprecision point of 0.70. Therefore, we decided to reduce the level of certainty by one level.

#### 3.3.2. Diagnostic Accuracy of the TB Score in Distinguishing Pulmonary TB From HC

A total of seven studies (3724 patients) evaluated the diagnostic accuracy of the TB score for distinguishing pulmonary TB from HC, finding a sensitivity of 0.75 (95% CI: 0.72–0.78; *I* ^2^ = 72*%*; low CoE), a specificity of 0.86 (95% CI: 0.79–0.90; *I* ^2^ = 89*%*; very low CoE) (Figure S5), an AUC of 0.87 (0.84–0.90) (Figure S6), a PPV of 0.90 (95% CI: 0.80–0.95; *I* ^2^ = 95*%*), an NPV of 0.66 (95% CI: 0.48–0.81; *I* ^2^ = 98*%*) (Figure S7), an LR+ of 4.52 (95% CI: 4.02–5.08), and an LR− of 0.30 (95% CI: 0.28–0.32) (Figure S8). The DOR was 17.78 (95% CI: 10.30–30.70; *I* ^2^ = 84*%*) (Figure S9).

In the hypothetical scenario of a 1000‐person cohort, 248 (238–257) patients may have been correctly identified as having pulmonary TB, whereas 82 (73–92) patients with pulmonary TB may have been misclassified as not having pulmonary TB. Additionally, 576 (529–603) people without pulmonary TB may have been correctly classified, whereas 94 (67–141) may have been misclassified as having TB (Table 3).

#### 3.3.3. Diagnostic Accuracy of the TB Score in Distinguishing Extrapulmonary Meningeal Tuberculosis From Other Neurological Diseases

A total of two studies (402 patients) evaluated the diagnostic accuracy of the TB score for distinguishing extrapulmonary meningeal TB from other neurological diseases, finding a sensitivity of 0.58 (95% CI: 0.52–0.65; *I* ^2^ = 9*%*; low CoE) and a specificity of 0.67 (95% CI: 0.46–0.83; *I* ^2^ = 91*%*; very low CoE) (Figure S10).

In the hypothetical scenario of a 1000‐person cohort, 191 (172–215) patients may have been correctly identified as having extrapulmonary meningeal TB, whereas 139 (115–158) patients with extrapulmonary meningeal TB may have been misclassified as not having extrapulmonary meningeal TB. Additionally, 449 (308–556) people without extrapulmonary meningeal TB may have been correctly classified, whereas 221 (114–362) may have been misclassified as having extrapulmonary meningeal TB (Table 3).

#### 3.3.4. Diagnostic Accuracy of the TB Score in Distinguishing Active TB From Latent TB

A total of four studies (1417 patients) evaluated the diagnostic accuracy of the TB score for distinguishing active from latent TB, finding a sensitivity of 0.73 (95% CI: 0.62–0.81; *I* ^2^ = 82*%*; very low CoE) and a specificity of 0.76 (95% CI: 0.68–0.83; *I* ^2^ = 81*%*; very low CoE) (Figure S11).

In the hypothetical scenario of a 1000‐person cohort, 241 (205–267) patients may have been correctly identified as having active TB, whereas 89 (63–125) patients with active TB may have been misclassified as not having active TB. Additionally, 509 (456–556) people without active TB may have been correctly classified, whereas 161 (114–214) may have been misclassified as having active TB (Table 3).

### 3.4. RoB and Applicability

Almost all studies had a low RoB across all domains. However, four studies had a high RoB in the domain of patient selection and applicability because they used a case–control design, and two other studies had an unclear RoB in the domain of reference test because they did not provide a detailed description of the test used (Figure 3 **)**.

**Figure 3:**
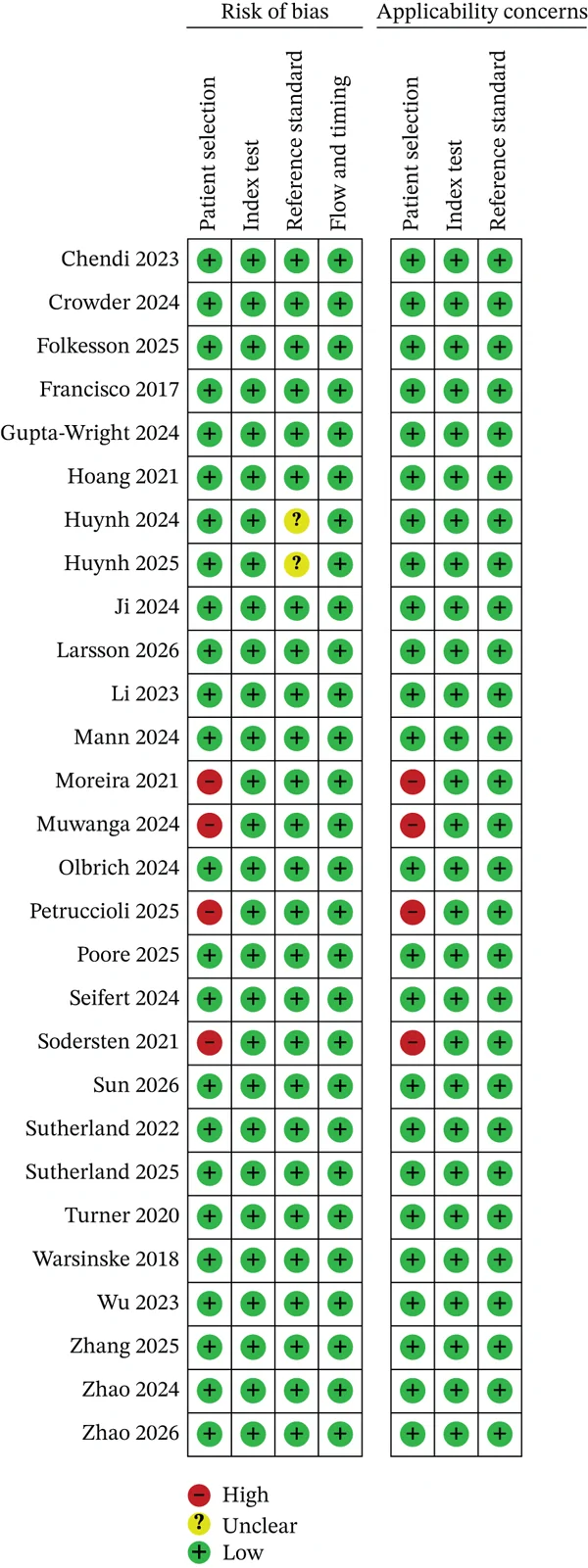
Graphical summary of the risk of bias and application concerns on included studies using the QUADAS‐2 tool.

### 3.5. Metaregression Analysis

Metaregression did not identify any study‐level covariates significantly associated with the sensitivity or specificity of the TB score for distinguishing pulmonary TB from ORD. Neither mean age, male sex, nor the proportion of patients with HIV or diabetes in the TB population showed a significant association with diagnostic sensitivity or specificity in crude or adjusted models (Table S5).

### 3.6. Sensitivity Analysis

Sensitivity analysis revealed that the sensitivity and specificity estimates remained stable across the leave‐one‐out analyses, with no substantial changes in the pooled estimates or heterogeneity when any individual study was sequentially excluded. (Figures S12 and S13).

### 3.7. Publication Bias

The Deeks plot showed a generally symmetrical distribution, and the Deeks regression asymmetry test did not suggest the presence of publication bias (multiple *R*‐squared = 0.0067, *p* = 0.7096) (Figure S14).

### 3.8. CoE

We considered a high initial CoE because all studies were of diagnostic test design. However, the certainty of evidence was downgraded primarily because of inconsistency and imprecision. For pulmonary TB versus ORD, both sensitivity and specificity were rated as having very low certainty, mainly because of substantial heterogeneity (*I* ^2^ > 60*%*) and imprecision. Similar concerns were observed across the other diagnostic comparisons, resulting in low to very low certainty of evidence. The CoE was low for sensitivity in pulmonary TB versus HC and meningeal tuberculosis versus other neurological diseases, whereas specificity in these comparisons and both outcomes for active versus latent TB were generally rated as very low. Therefore, although the pooled estimates provide evidence of the diagnostic performance of the TB score, confidence in these estimates remains limited (Table 3).

## 4. Discussion

In this review of 28 studies, the TB score showed moderate accuracy for distinguishing pulmonary TB from ORD, with a sensitivity of 0.80, specificity of 0.72, and an AUC of 0.82. Performance was slightly higher than HC (AUC 0.87) but decreased for extrapulmonary meningeal TB (sensitivity 0.58; specificity 0.67) and active versus latent TB (sensitivity 0.73; specificity 0.76). Overall, CoE was very low, and high heterogeneity remained unexplained.

The moderate diagnostic performance observed in our analysis for distinguishing pulmonary TB from ORD is like that described in other studies. Large validation studies with multiple cohorts of blood‐based transcriptomic signatures have reported an AUC that is nearly the same (0.84) [48] when comparing pulmonary TB to ORD and even slightly higher (0.89) [49] if HC patients were also included in the comparison, which may be consistent with the potential biological characteristics captured by the TB score, although the present evidence does not establish an inherent biological potential of the score. Similarly, at the clinical level, reviews evaluating alternative host biomarkers, such as PCR and multiprotein panels, have shown relatively high sensitivity (82%–84%), but variable specificity (67%–82%) in clinically relevant cohorts, such as seropositive populations [50, 51], suggesting that immune‐based diagnostics may face difficulties in distinguishing pulmonary TB from other ORDs. Furthermore, this difference in performance between pulmonary TB/ORD and pulmonary TB/HC could potentially be related to the fact that host‐response signatures may lose specificity in the presence of nontuberculous infections because of shared patterns of immune activation, particularly those related to IFN‐*γ* signaling and innate immune pathways [52]. Healthy individuals may have lower baseline immune activation, whereas ORD patients may exhibit inflammatory signatures that overlap with those associated with TB, potentially contributing to FPs.

On the other hand, the lower response observed in tuberculous meningitis could potentially be related to the compartmentalization of the immune response. In cases of pulmonary TB, the infection may induce a strong systemic response detectable in peripheral blood [53]; in contrast, extrapulmonary tuberculosis, especially that affecting the central nervous system, may be characterized by a more localized immune response, behind physiological barriers such as the blood–brain barrier [54]. Furthermore, the inflammatory profiles of tuberculous meningitis often overlap significantly with those of bacterial and viral meningitis, which could further contribute to lower specificity [55]. These mechanisms may provide possible explanations for the observed lower performance, but they cannot be established as causal explanations based on the available evidence.

The moderate performance in distinguishing active from latent tuberculosis may also be related to the biological complexity of the host response in these conditions. Latent tuberculosis is not a silent state but rather represents a dynamic balance between host immunity and bacterial persistence [56], often associated with intermittent immune activation. In this regard, it has been shown that individuals with latent TB who progress to active disease already exhibit elevated inflammatory markers even 1 month prior to diagnosis [57]. Furthermore, markers of immune activation significantly overlap between latent and active TB [58], which could potentially contribute to the difficulty in discriminating between these conditions. Overall, these biological mechanisms may contribute to the observed variability in diagnostic performance; however, the sources of heterogeneity could not be adequately identified with the available study‐level data. Therefore, these mechanisms should be considered hypotheses rather than established explanations for the observed diagnostic performance.

Regarding subgroups, age‐related differences (such as lower sensitivity in children) could potentially be associated with physiological and disease‐related characteristics; pediatric tuberculosis is typically paucibacillary and may exhibit differences in systemic inflammatory responses [59], which could potentially affect biomarker sensitivity. Conversely, the slightly higher accuracy in HIV‐positive populations could potentially be related to differences in systemic immune activation and interferon‐related pathways [60], rather than being interpreted as evidence of a biologically enhanced diagnostic signal. However, these subgroup differences were not statistically significant, and the underlying mechanisms therefore remain hypothetical.

Although the *GBP5*/*DUSP3*/*TBP* signature showed numerically higher sensitivity and the *GBP5*/*DUSP3*/*KLF2* signature showed numerically higher specificity, these differences were not statistically significant. *GBP5* and *DUSP3* are involved in interferon‐related inflammatory responses [61, 62], improving active disease detection, whereas *TBP* (a stable maintenance gene) enhances signal consistency [63]. In contrast, *KLF2* regulates immune responses and T‐cell trafficking, contributing to better discrimination between inflammatory states and improving specificity [64]. These biological functions could potentially provide a basis for the observed numerical differences between the signatures; however, the present analysis did not demonstrate statistically significant differences, and the available evidence does not allow a mechanistic explanation for these findings. Therefore, these observations should be considered hypothesis‐generating rather than evidence of differential biological performance between the *KLF2*‐ and *TBP*‐based signatures.

The findings suggest that the TB score may have a potential role within integrated diagnostic pathways, especially in resource‐limited settings requiring rapid, nonsputum‐based alternatives. However, pooled sensitivity remained below the minimum recommended threshold for a WHO triage test [65], and therefore, the available evidence does not support describing the TB score as a high‐sensitivity triage test or as a standalone tool for ruling out tuberculosis. Its potential role could instead be investigated as an adjunctive approach within diagnostic algorithms, particularly in combination with clinical assessment or other diagnostic tests. Future research should focus on prospective, real‐world cohorts with standardized reference criteria to define its potential role and added value within integrated diagnostic algorithms.

Although specificity approached the minimum WHO TPP threshold for a triage test, pooled sensitivity remained below the recommended minimum [65]; therefore, the TB score should not be considered a standalone triage replacement. However, this lack of consistency may depend on the selected threshold, patient population, and clinical context. Consequently, its potential role within the diagnostic pathway should be considered investigational rather than as an established strategy to rule out tuberculosis; specifically, within the current diagnostic protocol, the TB score could potentially be evaluated during the initial triage phase, following the clinical evaluation of patients with suspected tuberculosis and prior to microbiological confirmation. Furthermore, the TB score remains a promising approach among other available blood‐based host‐response biomarkers [13], especially given its nonsputum‐based nature. Nevertheless, further evidence is needed to determine its optimal role, including direct comparisons with other triage strategies, sequential testing algorithms, and robust cost‐effectiveness analyses.

## 5. Limitations and Strengths

This study has some limitations. First, most of the included studies were conducted in Asia and Africa and evaluated populations with a high TB burden, which limits the generalizability of our results. Second, the lack of certain variables, such as information on coinfections, prevented more subgroup analyses. Third, there was substantial variability in the cutoff values used across studies, and some studies did not report a cutoff. Differences in thresholds, together with variations in populations and assay implementation, may have contributed to heterogeneity in diagnostic performance and limit direct comparisons across studies. Fourth, the use of different reference standards, including culture and Xpert Ultra, may have introduced variability in the diagnostic accuracy estimates. Moreover, both methods have imperfect sensitivity and may result in misclassification of TB status, potentially affecting the diagnostic performance. Fifth, some studies also used case–control designs, which may overestimate diagnostic performance. Sixth, the absence of individual patient data limited the ability to perform robust analyses. Seventh, the search did not include Chinese language databases such as the China National Knowledge Infrastructure (CNKI), which may have resulted in the omission of relevant studies and introduced potential language or database selection bias. Eighth, information on loss to follow‐up and unevaluable samples was not consistently reported across studies, limiting our ability to assess whether these factors could have influenced the diagnostic accuracy estimates. Ninth, sensitivity and specificity were pooled separately, which does not account for the correlation between these measures and may not fully capture the overall diagnostic performance of the test. Finally, evidence was limited for certain clinical scenarios, particularly tuberculous meningitis and latent TB, which reduced the certainty in these groups. Despite these limitations, this study has several strengths. First, we evaluated the performance of the TB score for screening in health centers and included vulnerable populations in the subgroup analyses and metaregression. Second, we used culture or Xpert Ultra as reference standards while acknowledging their limitations, which allowed diagnostic performance to be assessed against commonly, used microbiological criteria. Finally, we conducted analyses of certainty of evidence and metaregression to further assess the diagnostic performance and explore potential sources of heterogeneity.

## 6. Conclusion

The TB score demonstrates moderate diagnostic accuracy for pulmonary tuberculosis, with pooled sensitivity and specificity below the levels required to support its use as a standalone triage or rule‐out test. Diagnostic performance varied across clinical scenarios, and substantial heterogeneity remained unexplained by the evaluated covariates. Although the TB score may have a potential role as an adjunct within diagnostic algorithms, this application remains investigational and requires further validation. Given the very low CoE and the high between‐study heterogeneity, its clinical utility remains uncertain, and prospective studies in representative populations are needed to establish its performance and potential role in tuberculosis diagnostic pathways.

## Author Contributions

Carlos Quispe‐Vicuña: Conceptualization, data curation, formal analysis, methodology, project administration, visualization, writing—original draft, and writing—review and editing. Dante Julca‐Marín: Conceptualization, data curation, methodology, writing—original draft, and writing—review and editing. Milagros Pascual‐Guevara: Conceptualization, data curation, methodology, and writing—review and editing. Fabian A. Chavez Ecos: Conceptualization, methodology, and writing—review and editing. Carlos J. Zumaran‐Nuñez: Conceptualization, methodology, and writing—review and editing. Fritz Fidel Váscones‐Román: Conceptualization, data curation, and writing—review and editing. Carlomagno Villarreal‐Inca: Conceptualization, methodology, project administration, supervision, and writing—review and editing. Elizabeth F. Hidalgo Ferrer: Conceptualization, methodology, project administration, supervision, and writing—review and editing. Miguel Cabanillas‐Lazo: Conceptualization, methodology, project administration, supervision, and writing—review and editing. Luz Quispe‐Garate: Conceptualization, methodology, project administration, supervision, and writing—review and editing. J. Smith Torres‐Roman: Conceptualization, methodology, project administration, supervision, and writing—review and editing. Julio A. Poterico: Conceptualization, methodology, project administration, supervision, and writing—review and editing. Miguel A. Arce‐Huamani: Conceptualization, methodology, project administration, supervision, and writing—review and editing. Vicente Benitez‐Zapata: Conceptualization, methodology, project administration, supervision, and writing—review and editing.

## Funding

No funding was received for this manuscript.

## Ethics Statement

The authors have nothing to report.

## Conflicts of Interest

The authors declare no conflicts of interest.

## Supporting information


**Supporting Information** Additional supporting information can be found online in the Supporting Information section. Table S1: PRISMA‐DTA 2020 Checklist. Table S2: Search strategy per searched electronic databases. Table S3: Agreement between reviewers during the independent review. Table S4: Excluded studies and reasons for exclusion of studies from the systematic review. Table S5: Metaregression models of diagnostic accuracy of TB score in distinguishing pulmonary TB from ORD. Figure S1: Summary receiver operating characteristic (sROC) of TB score in distinguishing pulmonary TB from ORD. Figure S2: (A) PPV and (B) NPV of TB score in distinguishing pulmonary TB from ORD. Figure S3: Fagan nomogram of TB score in distinguishing pulmonary TB from ORD for (A) LR+ and (B) LR−. Figure S4: Pooled diagnostic DOR of TB score in distinguishing pulmonary TB from ORD. Figure S5: (A) Sensitivity and (B) specificity of TB score in distinguishing pulmonary TB from HC. Figure S6: Summary receiver operating characteristic (sROC) of TB score in distinguishing pulmonary TB from HC. Figure S7: (A) PPV and (B) NPV of TB score in distinguishing pulmonary TB from HC. Figure S8: Fagan nomogram of TB score in distinguishing pulmonary TB from HC for (A) LR+ and (B) LR−. Figure S9: Pooled diagnostic DOR of TB score in distinguishing pulmonary TB from HC. Figure S10: (A) Sensitivity and (B) specificity of TB score in distinguishing extrapulmonary meningeal tuberculosis from other neurological diseases. Figure S11: (A) Sensitivity and (B) specificity of TB score in distinguishing active TB from passive TB. Figure S12: Sensitivity analysis of the (A) sensitivity and (B) specificity excluding each individual study in distinguishing pulmonary TB from ORD. Figure S13: Sensitivity analysis of the (A) sensitivity and (B) specificity excluding each individual study in distinguishing pulmonary TB from HC. Figure S14: Deeks plot of TB score in distinguishing PT from ORD.

## Data Availability

The data that support the findings of this study are available in the Supporting Information of this article.
