# Supplementary material for: Diagnostic Accuracy of the Three‐Gene Host‐Response Test in Blood for Tuberculosis: A Systematic Review
Source: Int J Microbiol. 2026 Nov 6;2026:3402245. doi: 10.1155/ijm/3402245 (PMC13743860; doi:10.1155/ijm/3402245)
Supplement: Supplementary file 1 [file IJM-2026-3402245-s001.docx]

**Diagnostic accuracy of the 3-gene host response test in blood for tuberculosis: A systematic review.**

**Supplementary material**

- **Table S1. PRISMA-DTA 2020 Checklist**
- **Table S2.** **Search strategy per searched electronic databases.**
- **Table S3. Agreement between reviewers during the independent review**
- **Table S4. Excluded studies and reasons for exclusion of studies from the systematic review**
- **Table S5. Meta-regression models of diagnostic accuracy of TB score in distinguishing pulmonary TB from ORD.**
- **Figure S1. Summary Receiver Operating Characteristic (sROC) of TB score in distinguishing pulmonary TB from ORD.**
- **Figure S2. (A) PPV and (B) NPV of TB score in distinguishing pulmonary TB from ORD.**
- **Figure S3. Fagan nomogram of TB score in distinguishing pulmonary TB from ORD for (A) LR+ and (B) LR-**
- **Figure S4. Pooled diagnostic DOR of TB score in distinguishing pulmonary TB from ORD.**
- **Figure S5. (A) Sensitivity and (B) Specificity of TB score in distinguishing pulmonary TB from HC.**
- **Figure S6. Summary Receiver Operating Characteristic (sROC) of TB score in distinguishing pulmonary TB from HC.**
- **Figure S7. (A) PPV and (B) NPV of TB score in distinguishing pulmonary TB from HC**
- **Figure S8. Fagan nomogram of TB score in distinguishing pulmonary TB from HC for (A) LR+ and (B) LR-**
- **Figure S9. Pooled diagnostic DOR of TB score in distinguishing pulmonary TB from HC.**
- **Figure S10. (A) Sensitivity and (B) Specificity of TB score in distinguishing extrapulmonary meningeal tuberculosis from other neurological diseases**
- **Figure S11. (A) Sensitivity and (B) Specificity of TB score in distinguishing active TB from passive TB**
- **Figure S12. Sensitivity analysis in distinguishing pulmonary TB from ORD. Sensitivity (A) and Specificity (B)**
- **Figure S13. Sensitivity analysis in distinguishing pulmonary TB from HC. Sensitivity (A) and Specificity (B)**
- **Figure S14. Deeks plot of TB score in distinguishing pulmonary TB from ORD.**

## Table S1. PRISMA-DTA 2020 Checklist

| **Section/topic** | **#** | **PRISMA-DTA Checklist Item** | **Reported on page #** |
| --- | --- | --- | --- |
| **TITLE / ABSTRACT** | | |  |
| Title | 1 | Identify the report as a systematic review (+/- meta-analysis) of diagnostic test accuracy (DTA) studies. | 1 |
| Abstract | 2 | Abstract: See PRISMA-DTA for abstracts. | 2 |
| **INTRODUCTION** | | |  |
| Rationale | 3 | Describe the rationale for the review in the context of what is already known. | 3 |
| Clinical role of index test | D1 | State the scientific and clinical background, including the intended use and clinical role of the index test, and if applicable, the rationale for minimally acceptable test accuracy (or minimum difference in accuracy for comparative design). | 3 |
| Objectives | 4 | Provide an explicit statement of question(s) being addressed in terms of participants, index test(s), and target condition(s). | 3 |
| **METHODS** | | |  |
| Protocol and registration | 5 | Indicate if a review protocol exists, if and where it can be accessed (e.g., Web address), and, if available, provide registration information including registration number. | 4 |
| Eligibility criteria | 6 | Specify study characteristics (participants, setting, index test(s), reference standard(s), target condition(s), and study design) and report characteristics (e.g., years considered, language, publication status) used as criteria for eligibility, giving rationale. | 4 |
| Information sources | 7 | Describe all information sources (e.g., databases with dates of coverage, contact with study authors to identify additional studies) in the search and date last searched. | 4 and Supplemental material |
| Search | 8 | Present full search strategies for all electronic databases and other sources searched, including any limits used, such that they could be repeated. | 4 and Supplemental |
| Study selection | 9 | State the process for selecting studies (i.e., screening, eligibility, included in systematic review, and, if applicable, included in the meta-analysis). | 4 |
| Data collection process | 10 | Describe method of data extraction from reports (e.g., piloted forms, independently, in duplicate) and any processes for obtaining and confirming data from investigators. | 4-5 |
| Definitions for data extraction | 11 | Provide definitions used in data extraction and classifications of target condition(s), index test(s), reference standard(s) and other characteristics (e.g. study design, clinical setting). | 5 |
| Risk of bias and applicability | 12 | Describe methods used for assessing risk of bias in individual studies and concerns regarding the applicability to the review question. | 5 |
| Diagnostic accuracy measures | 13 | State the principal diagnostic accuracy measure(s) reported (e.g. sensitivity, specificity) and state the unit of assessment (e.g. per-patient, per-lesion). | 5 |
| Synthesis of results | 14 | Describe methods of handling data, combining results of studies and describing variability between studies. This could include, but is not limited to: a) handling of multiple definitions of target condition. b) handling of multiple thresholds of test positivity, c) handling multiple index test readers, d) handling of indeterminate test results, e) grouping and comparing tests, f) handling of different reference standards | 5-6 |

| **Section/topic** | **#** | **PRISMA-DTA Checklist Item** | **Reported on page #** |
| --- | --- | --- | --- |
| Meta-analysis | D2 | Report the statistical methods used for meta-analyses, if performed. | 5-6 |
| Additional analyses | 16 | Describe methods of additional analyses (e.g., sensitivity or subgroup analyses, meta-regression), if done, indicating which were pre-specified. | 5-6 |
| **RESULTS** | | |  |
| Study selection | 17 | Provide numbers of studies screened, assessed for eligibility, included in the review (and included in meta-analysis, if applicable) with reasons for exclusions at each stage, ideally with a flow diagram. | 6-7 and Figure 1 |
| Study characteristics | 18 | For each included study provide citations and present key characteristics including: a) participant characteristics (presentation, prior testing), b) clinical setting, c) study design, d) target condition definition, e) index test, f) reference standard, g) sample size, h) funding sources | 6-7 and Table 1 |
| Risk of bias and applicability | 19 | Present evaluation of risk of bias and concerns regarding applicability for each study. | 8 and Figure 2 |
| Results of individual studies | 20 | For each analysis in each study (e.g. unique combination of index test, reference standard, and positivity threshold) report 2x2 data (TP, FP, FN, TN) with estimates of diagnostic accuracy and confidence intervals, ideally with a forest or receiver operator characteristic (ROC) plot. | 7-8 |
| Synthesis of results | 21 | Describe test accuracy, including variability; if meta-analysis was done, include results and confidence intervals. | 7-8 |
| Additional analysis | 23 | Give results of additional analyses, if done (e.g., sensitivity or subgroup analyses, meta-regression; analysis of index test: failure rates, proportion of inconclusive results, adverse events). | 7 and Table 2 |
| **DISCUSSION** | | |  |
| Summary of evidence | 24 | Summarize the main findings including the strength of evidence. | 9 |
| Limitations | 25 | Discuss limitations from included studies (e.g. risk of bias and concerns regarding applicability) and from the review process (e.g. incomplete retrieval of identified research). | 11 |
| Conclusions | 26 | Provide a general interpretation of the results in the context of other evidence. Discuss implications for future research and clinical practice (e.g. the intended use and clinical role of the index test). | 11 |
| **FUNDING** | | |  |
| Funding | 27 | For the systematic review, describe the sources of funding and other support and the role of the funders. | 12 |

*Adapted From:* McInnes MDF, Moher D, Thombs BD, McGrath TA, Bossuyt PM, The PRISMA-DTA Group (2018). Preferred Reporting Items for a Systematic Review and Meta-analysis of Diagnostic Test Accuracy Studies: The PRISMA-DTA Statement. JAMA. 2018 Jan 23;319(4):388-396. doi: 10.1001/jama.2017.19163.

## Table S2. Search strategy per searched electronic databases.

| Database | Search strategy | Results |
| --- | --- | --- |
| Pubmed | #1: "Cepheid 3-gene Host"[TIAB] OR "MTB-HR"[TIAB] OR "cepheid xpert"[TIAB] OR "cepheid genexpert"[TIAB] OR "TB score"[TIAB]  #2: "Tuberculosis"[Mesh] OR Tuberculos*[TIAB] OR “Kochs Disease”[TIAB] OR “Koch's Disease”[TIAB] OR “Koch Disease”[TIAB]  #3: #1 AND #2 | 129 |
| Scopus | #1: TITLE-ABS-KEY("Cepheid 3-gene Host" OR MTB-HR OR "cepheid xpert" OR "cepheid genexpert" OR “TB score”)  #2: TITLE-ABS-KEY(Tuberculos* OR “Kochs Disease” OR “Koch's Disease” OR “Koch Disease”)  #3: #1 AND #2 | 155 |
| WOS | #1: TS=("Cepheid 3-gene Host" OR MTB-HR OR "cepheid xpert" OR "cepheid genexpert" OR “TB score”) OR TI=("Cepheid 3-gene Host" OR MTB-HR OR "cepheid xpert" OR "cepheid genexpert" OR “TB score”) OR AK=("Cepheid 3-gene Host" OR MTB-HR OR "cepheid xpert" OR "cepheid genexpert" OR “TB score”) OR AB=("Cepheid 3-gene Host" OR MTB-HR OR "cepheid xpert" OR "cepheid genexpert" OR “TB score”) OR KP=("Cepheid 3-gene Host" OR MTB-HR OR "cepheid xpert" OR "cepheid genexpert" OR “TB score”)  #2: TS=(Tuberculos* OR “Kochs Disease” OR “Koch's Disease” OR “Koch Disease”) OR TI=(Tuberculos* OR “Kochs Disease” OR “Koch's Disease” OR “Koch Disease”) OR AK=(Tuberculos* OR “Kochs Disease” OR “Koch's Disease” OR “Koch Disease”) OR KP=(Tuberculos* OR “Kochs Disease” OR “Koch's Disease” OR “Koch Disease”) OR AB=(Tuberculos* OR “Kochs Disease” OR “Koch's Disease” OR “Koch Disease”)  #3: #1 AND #2 | 124 |
| Embase | #1: ('cepheid xpert'/exp OR 'cepheid xpert' OR 'cepheid genexpert'/exp OR 'cepheid genexpert' OR 'mtb-hr' OR 'cepheid 3-gene host' OR 'TB score')  #2: ('tuberculosis'/exp OR 'koch`s disease':ti,ab,kw OR 'm. tuberculosis infection':ti,ab,kw OR 'mycobacterium tuberculosis infection':ti,ab,kw OR 'tb (tuberculosis)':ti,ab,kw OR 'tb case':ti,ab,kw OR 'tb cases':ti,ab,kw OR 'tb disease':ti,ab,kw OR 'tb infection':ti,ab,kw OR 'active tb':ti,ab,kw OR 'active tuberculosis':ti,ab,kw OR 'case of tb':ti,ab,kw OR 'cases of tb':ti,ab,kw OR 'chronic tuberculosis':ti,ab,kw OR 'infection by m. tuberculosis':ti,ab,kw OR 'infection by mycobacterium tuberculosis':ti,ab,kw OR 'infection due to m. tuberculosis':ti,ab,kw OR 'infection due to mycobacterium tuberculosis':ti,ab,kw OR 'infection of m. tuberculosis':ti,ab,kw OR 'infection of mycobacterium tuberculosis':ti,ab,kw OR 'minimal tuberculosis':ti,ab,kw OR 'minimum tuberculosis':ti,ab,kw OR 'tuberculosis':ti,ab,kw OR 'tuberculous infection':ti,ab,kw OR 'tuberculous lesion':ti,ab,kw)  #3: #1 AND #2 | 188 |
| Cochrane Central | #1: ("Cepheid 3-gene Host" OR MTB-HR OR "cepheid xpert" OR "cepheid genexpert" OR “TB score”):ti,ab,kw  #2: (Tuberculos* OR “Kochs Disease” OR “Koch's Disease” OR “Koch Disease”):ti,ab,kw  #3: #1 AND #2 | 20 |
| Google Scholar | "Cepheid 3-gene Host" AND tuberculosis | Only first 100 results |
| LILACS | #1: ("Cepheid 3-gene Host" OR MTB-HR OR "cepheid xpert" OR "cepheid genexpert" OR “TB score”):title,resume,abstract  #2: (Tuberculos* OR “Kochs Disease” OR “Koch's Disease” OR “Koch Disease”):title,resume,abstract  #3: #1 AND #2 | 6 |

## Table S3. Agreement between reviewers during the independent review

| **STAGE** | **PRELIMINARY RESULTS** | | | | | | | **FINAL RESULTS** | |
| --- | --- | --- | --- | --- | --- | --- | --- | --- | --- |
|  | **N° of records** | **Included by both** | **Excluded by both** | **Included only by first reviewer** | **Included only by second reviewer** | **% agreement** | **Cohen's Kappa coefficient** | **Included** | **Excluded** |
|  | 722 |  |  |  |  |  |  | 28 | 694 |
| **First calibration** | 20 | 12 | 7 | 1 | 0 | 95 | 0.93 | 13 | 7 |
| **Second calibration** | 20 | 13 | 6 | 1 | 0 | 95 | 0.94 | 14 | 6 |
| **Review of titles and abstracts** | 522 | 41 | 474 | 2 | 5 | 98.7 | 0.94 | 43 | 479 |
| **Review of full texts** | 43 | 27 | 15 | 1 | 0 | 97.7 | 0.95 | 28 | 15 |

## Table S4. Excluded studies and reasons for exclusion of studies from the systematic review

| **AUTHOR** | **YEAR** | **TÍTULO** | **REASON FOR EXCLUSION** |
| --- | --- | --- | --- |
| Mendelsohn | 2022 | Prospective multicentre head-to-head validation of host blood transcriptomic biomarkers for pulmonary tuberculosis by real-time PCR | Diagnostic accuracy was not evaluated |
| Nakiboneka | 2024 | Specific human gene expression in response to infection is an effective marker for diagnosis of latent and active tuberculosis | The population was genetically sampled |
| Yao | 2022 | Whole blood GBP5 protein levels in patients  with and without active tuberculosis | The TB score was not assessed |
| Zhang | 2024 | A blood-based 3-gene signature score for therapeutic monitoring in patients with pulmonary tuberculosis | Diagnostic accuracy was not evaluated |
| Sweetser | 2025 | A Prospective Evaluation of a Three-Gene Host Response Signature to Classify Tuberculosis Severity in Children | Diagnostic accuracy was not evaluated |
| Sweeney | 2016 | Genome-wide expression for diagnosis of pulmonary tuberculosis: a multicohort analysis | The population was genetically sampled |
| Stanciu | 2025 | Physiologic-Inflammatory-Nutrition (TRIAD-TB) Score at 72 Hours Predicts 30-Day Mortality and Length of Stay in Pulmonary Tuberculosis: A Prospective Cohort Study | Diagnostic accuracy was not evaluated |
| Sweetser | 2026 | Evaluating the Cepheid Xpert TB/LTBI research-use-only assay for detection of active and latent Mycobacterium tuberculosis infection | The TB score was not assessed |
| Paul | 2025 | Comparative assessment of cartridge based nucleic acid amplification technique (CBNAAT) with gold standard LJ culture and smear microscopy in diagnosis of pulmonary and extra pulmonary tuberculosis in Tripura. | The TB score was not assessed |
| Bhatia | 2025 | Trends in TB scores and efficiency of molecular diagnostic methods across key districts of Madhya Pradesh | The TB score was not assessed |
| Ahirwar | 2025 | Estimate of TB incidence and a critical analysis of programmatic data of TB score from Sub national Certification survey of district Niwari, M.P., India. | The TB score was not assessed |
| Zhao | 2025 | [Construction of etiological diagnosis model for pathogen-negative pulmonary tuberculosis using tuberculosis scores of GBP5, DUSP3, and TBP genes combined with inflammatory factors] | No full text |
| Drane | 2025 | Non-sputum-based triage and confirmatory diagnostic tests for pediatric TB. | The TB score was not assessed |
| Klinkenberg | 2025 | Lessons Learned from Early Implementation and Scale-up of Stool-Based Xpert Testing to Diagnose Tuberculosis in Children. | The TB score was not assessed |
| Abdulgader | 2026 | Accuracy and clinical utility of near-point-of-care blood tests for predicting incident tuberculosis in exposed contacts in high burden settings: a multi-country observational cohort study | Diagnostic accuracy was not evaluated |

**Table S5. Meta-regression models of diagnostic accuracy** **of TB score in distinguishing pulmonary TB from ORD.**

| **Variables** | **n studies** | **Crude** | | | **Adjusted** | | |
| --- | --- | --- | --- | --- | --- | --- | --- |
|  |  | **OR** | **95% CI** | ***p*** | **OR** | **95% CI** | ***p*** |
| **Sensitivity** | | | | | | | |
| Mean age (years) | 22 | 1.01 | 0.99–1.04 | 0.301 | 1.02 | 0.99–1.06 | 0.262 |
| Sex male (%) | 23 | 1.00 | 0.97–1.03 | 0.842 | 1.01 | 0.97–1.04 | 0.702 |
| HIV in TB population (%) | 18 | 1.01 | 0.99–1.02 | 0.325 | 1.01 | 0.99–1.03 | 0.224 |
| DM in TB population (%) | 7 | 0.96 | 0.85–1.07 | 0.440 | – | – | – |
| **Specificity** | | | | | | | |
| Mean age (years) | 22 | 1.01 | 0.98–1.04 | 0.539 | 1.01 | 0.97–1.05 | 0.626 |
| Sex male (%) | 23 | 0.99 | 0.96–1.02 | 0.561 | 0.98 | 0.94–1.02 | 0.330 |
| HIV in TB population (%) | 18 | 1.00 | 0.98–1.01 | 0.478 | 0.99 | 0.97–1.01 | 0.314 |
| DM in TB population (%) | 7 | 0.99 | 0.96–1.02 | 0.558 | – | – | – |
| OR: Odds ratio. In bold significant p-values (p<0.05). | | | | | | | |

## Figure S1. Summary Receiver Operating Characteristic (SROC) curve of TB score in distinguishing pulmonary TB from ORD.

## Figure S2. (A) PPV and (B) NPV of TB score in distinguishing pulmonary TB from ORD.

## Figure S3. Fagan nomogram of TB score in distinguishing pulmonary TB from ORD for (A) LR+ and (B) LR-

## Figure S4. Pooled diagnostic DOR of TB score in distinguishing pulmonary TB from ORD.

## Figure S5. (A) Sensitivity and (B) Specificity of TB score in distinguishing pulmonary TB from HC.

## Figure S6. Summary Receiver Operating Characteristic (sROC) of TB score in distinguishing pulmonary TB from HC.

## Figure S7. (A) PPV and (B) NPV of TB score in distinguishing pulmonary TB from HC

## Figure S8. Fagan nomogram of TB score in distinguishing pulmonary TB from HC for (A) LR+ and (B) LR-

## Figure S9. Pooled diagnostic DOR of TB score in distinguishing pulmonary TB from HC.

## Figure S10. (A) Sensitivity and (B) Specificity of TB score in distinguishing extrapulmonary meningeal tuberculosis from other neurological diseases

## Figure S11. (A) Sensitivity and (B) Specificity of TB score in distinguishing active TB from passive TB

## Figure S12. Sensitivity analysis in distinguishing pulmonary TB from ORD. Sensitivity (A) and Specificity (B)

## Figure S13. Sensitivity analysis in distinguishing pulmonary TB from HC. Sensitivity (A) and Specificity (B)

## Figure S14. Deeks plot of TB score in distinguishing pulmonary TB from ORD.

Multiple R-squared: 0.0067, p-value: 0.7096
